# Construction of tandem diabody (IL-6/CD20)-secreting human umbilical cord mesenchymal stem cells and its experimental treatment on diffuse large B cell lymphoma

**DOI:** 10.1186/s13287-022-03169-4

**Published:** 2022-09-14

**Authors:** Jiayi Zhang, Minglu Zhong, Weijie Zhong, Yanfei Lan, Zhaohu Yuan, Yuyou Duan, Yaming Wei

**Affiliations:** 1 Department of Blood Transfusion, The Second Affiliated Hospital, School of Medicine, South China University of Technology, Guangzhou, Guangdong China; 2 Guangdong Engineering Research Center of Precise Transfusion, Guangzhou, Guangdong China; 3 Department of Geriatrics, Hematology and Oncology Ward, the Second Affiliated Hospital, School of Medicine, South China University of Technology, Guangzhou, Guangdong China; 4 Laboratory of Stem Cells and Translational Medicine, Institutes for Life Sciences and School of Medicine, South China University of Technology, Guangzhou, Guangdong China; 5 National Engineering Research Center for Tissue Restoration and Reconstruction, South China University of Technology, Guangzhou, Guangdong China; 6 Key Laboratory of Biomedical Engineering of Guangdong Province, South China University of Technology, Guangzhou, Guangdong China

**Keywords:** Tandem diabody, Mesenchymal stem cells, Diffuse large B cell lymphoma, IL-6 antibody, CD20 antibody

## Abstract

**Background:**

More than 40% patients with diffuse large B cell lymphoma (DLBCL) experienced relapse or refractory (R/R) lymphoma after the standard first R-CHOP therapy. IL-6 was reportedly associated with chemotherapy resistance of rituximab. Further, mesenchymal stem cells (MSCs) are known as the potential cell vehicle for their tropism toward tumor. A MSCs-based tandem diabody for treating DLBCL is currently lacking.

**Methods:**

We constructed a tandem diabody (Tandab(IL-6/CD20)) with modified umbilical cord MSCs (UCMSCs) and designed a cell-based Tandab releasing system. Western blot, qPCR and immunofluorescence were used to confirm the construction and expression of lentivirus-infected UCMSCs. The vitality, apoptosis and homing abilities of UCMSCs were examined via CCK-8 assay, apoptosis, wound healing and migration analysis. Cell binding assay was used to demonstrate the targeting property of Tandab binding to CD20-positive DLBCL cells. Furthermore, we evaluated the viability of SU-DHL-2 and SU-DHL-4 by using CCK-8 and EDU assay after the treatment of UCMSCs-Tandab(IL-6/CD20).

**Results:**

Tandab protein peaked at 6273 ± 487 pg/ml in the medium on day 7 after cell culture. The proliferation and homing ability of UCMSCs did not attenuate after genetically modification. Immunofluorescence images indicated the Tandab protein bound to the lymphoma cells. UCMSCs-Tandab(IL-6/CD20) inhibited the growth of SU-DHL-2 or SU-DHL-4 cells in vitro.

**Conclusions:**

UCMSCs-Tandab(IL-6/CD20), which bound with both tumor-associated surface antigens and pro-tumor cytokines in tumor microenvironment, might serve as a potential treatment for DLBCL, evidenced by inhibiting the growth of SU-DHL-2 or SU-DHL-4 cells.

**Supplementary Information:**

The online version contains supplementary material available at 10.1186/s13287-022-03169-4.

## Background

Lymphoma, an aggressive malignancy that originates from lymphoid system, accounts for 3.2% incidence and 2.8% mortality rate in all new cancer cases and deaths worldwide [1]. Non-Hodgkin’s lymphoma (NHL) represents approximately 90% of all lymphoma, while diffuse large B cell lymphoma (DLBCL) is the most common subtype in NHL, accounting for 42.5% [2]. The first standard regimen R-CHOP (rituximab, cyclophosphamide, doxorubicin, vincristine and prednisone) presents a promising efficacy in the treatment of DLBCL [3]. However, it is reported that more than 40% of patients suffered from relapse or refractory (R/R) lymphoma following immunochemotherapy, with a lack of standard second-line therapy [4]. Hence, it is of great importance to develop a novel and more tailored therapeutic regimen for patients with R/R DLBCL.

IL-6, a pleiotropic cytokine, is associated with various biological responses, including inflammation, immunity, metabolism, angiogenesis and neural processes [5, 6]. Recent studies have shown that IL-6 plays an important role in tumor progression in terms of tumor cell proliferation, metastasis and drug resistance [7–10]. Our previous studies demonstrated that IL-6 in tumor microenvironment (TME) is closely related to the growth of DLBCL [7, 11]. We have found that IL-6 induces chemotherapy resistance for rituximab (anti-CD20 monoclonal antibody), protects lymphoma cells from spontaneous and drug-induced apoptosis, and eventually promotes the growth of DLBCL through JAK/STAT3 and PI3K/Akt pathway [7, 11]. Other studies have also proved that high level of IL-6 is implemented as a potential negative biomarker for the growth of hematologic malignancies like Hodgkin’s lymphoma (HL) [8], extranodal natural killer T cell lymphoma (ENKTL) [9] and mantle cell lymphoma (MCL) [10]. Neutralizing IL-6 or blocking IL-6 receptors would inhibit IL-6/IL-6 receptor axis, abrogate the protection of tumor cells from spontaneous- and drug-induced apoptosis, and improve the cellular sensitivity to chemotherapy agents [5, 7, 10].

MSCs, which could be isolated from bone marrow (BM), umbilical cord (UC), Wharton’s jelly, adipose tissue, placenta or skeletal tissues, are implemented as a promising candidate in cell-based therapy. The Mesenchymal and Tissue Stem Cell Committee of the International Society for Cellular Therapy had proposed the minimal criteria to define human MSC [12–14]. First, MSCs must be purified from the BM stromal population and be plastic adherent based under standard culture conditions. Second, MSC must express CD105, CD73 and CD90 and lack expression of CD45, CD34, CD14 or CD11b, CD79a or CD19 and HLA-DR surface molecules. Third, MSC could differentiate into osteoblasts, adipocytes and chondroblasts in vitro. Compared with other MSCs, UCMSCs have been regarded as a superior source for its ease to harvest, low immunogenicity and innate homing ability [15–17]. Previous studies have proven that the expansion and differentiation abilities of MSCs derived from other sources decrease significantly with age, whereas UCMSCs possess prolonged culture period and high proliferation capacity [18]. Moreover, preclinical studies and clinical trials have proven that MSCs could produce therapeutic agents by means of genetic modification [19].

It is reported that monotherapy with rituximab tends to develop drug resistance in the treatment of lymphoma. Based upon the fact that MSCs could express antibodies in targeting multiple receptors on the surface of tumor cells and tumor-promoting factors in tumor microenvironment (TME) [20, 21], in this study we designed to establish a Tandem diabody and construct UCMSCs-Tandab(IL-6/CD20) and validated its therapeutic effect on DLBCL. Therefore, this study aimed to construct a cell-based releasing system that could locally and persistently express CD20 and IL-6 fusion antibody (Tandab(IL-6/CD20)) and to explore the therapeutic potential of UCMSCs-Tandab(IL-6/CD20) in DLBCL cells.

## Methods

### Cell culture

UCMSCs and SU-DHL-2 (ABC subtype)/SU-DHL-4 (GCB subtype) were obtained from Cyagen Biosciences (HUXUC-01001, China) and EK-Bioscience (CC-Y1678/CC-Y1712, China), respectively. Human peripheral blood mononuclear cells (PBMCs) from healthy donors were separated using density gradient centrifugation with Lymphoprep (00,118, STEMCELL Technologies, Canada). UCMSCs were cultured in Human Umbilical Cord Mesenchymal Stem Cell complete medium (HUXUC-90011, Cyagen Biosciences, China) containing 10% FBS. SU-DHL-2, SU-DHL-4 and purified PBMCs were maintained in RPMI 1640 medium (C11875500BT, Gibco, The USA), supplemented with 10% FBS.

### Construction of lentivirus and transduction of UCMSCs

We have designed a Tandab vector that contained fusion Tandab(IL-6/CD20) fragment, green fluorescent protein (GFP) cDNA and hexa-histidine (6 × His) tag; empty vector with GFP cDNA was regarded as negative control. Both vectors were entrusted to Guangzhou Yuanjing Company (Ubigene, China) for synthesis. Tandab gene was amplified and subcloned into the lentivirus vector YOE-LV001 to produce lentivirus vector YOE-LV001-Tandab (Tandab(CD20/IL-6)-6 × His-GFP vector). The Tandab virus was produced from the transient transfection of the HEK293T cells (Ubigene, China) with Tandab(CD20/IL-6)-6 × His-GFP vector together with pCMV-VSVG envelope vector and psPAX2 packaging vector (Ubigene, China). Before the transduction, UCMSCs at the logarithmic growth phase were digested into single-cell suspension, seeded in a 6-well culture plate and cultured in a humidified incubator at 37℃ with 5% CO^2^. When the confluence reached 30–50%, lentivirus virus (Tandab virus and Empty virus) (MOI = 10) was added to the culture medium, respectively, and polybrene was supplemented to promote the infection. Subsequently, the culture medium was removed and changed with fresh medium that contained 1.5 μg/ml puromycin for selecting genetically engineered UCMSCs. The genetically engineered UCMSCs were named as UCMSCs-Tandab(IL-6/CD20) and UCMSCs-Vector, respectively. The ratio of two kinds of lentivirus-infected UCMSCs was observed under an inverted fluorescence microscope (IX71, Olympus, Japan).

### Characterization of UCMSCs

In light of the guidelines established by the International Society for Cellular Therapy, MSCs express stromal markers CD29, CD73, CD90 and CD105 (≥ 90%), without expression of CD11b, CD14, CD34, CD45 and HLA-DR (≤ 2%) [14]. 8 × 10^5^ UCMSCs-Tandab(IL-6/CD20), UCMSCs-Vector and UCMSCs at logarithmic phase were, respectively, trypsinized and incubated with PE-conjugated or PerCP cy5.5-conjugated mouse anti-human CD34, CD90 and CD105 antibodies, which were subsequently used to stain cells and analyze with flow cytometer (C6, BD Biosciences, The USA). The expression levels were demonstrated in a histogram graph by using a FlowJo software (10.5.3, The USA).

### Cell proliferation assay

CCK-8 assay was performed to assess the proliferation of UCMSCs-Tandab(IL-6/CD20) and UCMSCs-Vector. Cells were seeded in a 96-well plate at a density of 2 × 10^3^ cells. On days 1, 2, 3, 4, and 5, 10 ul CCK-8 reagent (C0043, Beyotime, China) was added to each well and incubated at 37℃ for 4 h, the optical density (OD) value with absorbance at 450 nm was measured via a Microplate Reader (Cytation 5, Bio-Tek, The USA).

### Cell apoptosis assay

It was previously reported that early and late apoptotic cells were marked as Annexin V (Annexin +) and 7-AAD (7-AAD +) positive, respectively [22]. Hence, the Annexin V-APC and 7-AAD staining solution (KGA1026, keyGEN, China) were used to assess the apoptosis in 1 × 10^5^ cells from both the UCMSCs-Tandab(IL-6/CD20) and UCMSCs-Vector. Annexin V expression was detected with a flow cytometer (C6, BD Biosciences, The USA) in APC channel, exhibiting early and late apoptosis, respectively.

### Cell migration assay

Wound healing scratch assay and transwell migration assay were used to analyze the cell migration ability. For wound healing scratch assay, all three UCMSC cell lines were seeded into triplicate wells of 6-well plate (5 × 10^5^cells/well) and cultured until reaching 90% confluency. Subsequently, artificial scratch wound was drawn with a sterile pipette tip at the center of each well. Wound distances were photographed at 0, 12, and 24 h by using a microscope (IX71, Olympus, Japan).

As described in Yan’s study on transwell migration assay [23], the UCMSC cell lines were seeded in the upper chamber of a 24-well transwell plate with serum-free DMEM basic culture medium (C11885500BT, Gibco, The USA) and SU-DHL-4 cells were cultured in the lower chamber that supplemented with complete culture medium containing 10% FBS. Cell-free medium was defined as a control group. After cultured for 24 h at 37 ℃, residual cells on the upper surface were wiped off, fixed and stained. The number of stained UCMSCs cells was observed and counted under a microscope (IX71, Olympus, Japan).

### RNA extraction and quantitative real-time PCR

The expression level of Tandab mRNA in UCMSCs cell lines was detected by qRT-PCR. Total RNA was extracted from UCMSCs-Tandab(IL-6/CD20), UCMSCs-Vector and UCMSCs by using RNAiso Plus reagent (9109, Takara, Japan), and the RNA measurement was conducted with a NanoDrop micro-spectrophotometer (Thermo Fisher Scientific, The USA). The synthesis of cDNA was performed by using RT Master Mix reagent (RR036A, Takara, Japan).

The relative expression level of Tandab was evaluated using SYBR Green Master Mix reagent (A25742, Thermo Fisher Scientific, The USA) in a Quant StudioTM 1 Real-Time PCR System (Thermo Fisher Scientific, The USA); β-actin was used as the reference gene. The primers were as follows: Tandab: Forward 5’-GCAAACTAATGAGGCTCCGC-3’, Reverse 5’-CTTCACCCAGTGCATGTTGTAG-3’, *β*-actin: Forward 5’-CCAGCCATGTACGTTGCTATC-3’, Reverse 5’-CTTAATGTCACGCACGATTTCC-3’. The condition of each PCR reaction was as follows: 50℃ for 2 min, 95 ℃ for 2 min, followed by 40 cycles of denaturation at 95℃ for 3 s and annealing/extending at 60℃ for 30 s. The mean value was calculated, and the expression values were analyzed by 2-ΔΔCt method.

### Western blot

To detect the expression of Tandab fusion protein, as reported in Adibkia’s study [24], UCMSCs-Tandab(IL-6/CD20), UCMSCs-Vector and UCMSCs were cultured in 6-well plates for 4 days; subsequently, the supernatants and cell lysates were harvested. Protein samples were loaded on 10% SDS-PAGE gels and electrophoresed and then transferred on PVDF membranes. Additionally, membranes were exposed to primary antibodies (rabbit anti-human His-Tag (12698S, Cell Signaling Technology, The USA) and rabbit anti-human GAPDH (10,494–1-AP, Proteintech, The USA) antibodies overnight. Afterward, the membranes were incubated with a goat anti-rabbit horseradish peroxidase (HRP)-conjugated secondary antibody (ab205718, Abcam, The USA) for 1 h. Afterward, the bands were visualized by using ECL western blotting detection substrate in a chemiluminescent imaging system.

### ELISA

The secretion of UCMSCS-Tandab (IL-6/CD20) at different time points was measured by ELISA. After transduction, the supernatants of UCMSCs-Tandab(IL-6/CD20) were collected every day and the expression of Tandab protein was assessed by using a His Tag ELISA Detection kit (L00436, GenScript, The USA) according to the manufacturer's protocol. OD values were measured at 450 nm in a Microplate Reader (Cytation 5, Bio-Tek, The USA), and the absorbance readings were converted to concentrations.

### Immunofluorescence

The expression of target protein labeled with 6 × His Tag in UCMSCs-Tandab(IL-6/CD20) was detected by immunofluorescence; UCMSCs-Vector and UCMSCs were defined as the negative control. Cells were cultured in 35-mm confocal dishes, respectively. When 70–80% confluence was reached, the cells were fixed and incubated with His-Tag Rabbit primary monoclonal antibody at 4℃ overnight, followed by incubation with Alexa Fluor® 594 conjugate goat anti-rabbit secondary antibodies (#8889, Cell Signaling Technology, The USA) at room temperature for 45 min. After washing three times, the nuclei were stained with DAPI staining solution for 3 min. The images were detected and captured in a single photon confocal microscopy (Ti-E A1, Nikon, Japan).

### Cell binding assay

On the basis of Zhang’s study [25], cell binding assay was performed to analyze the binding activity of Tandab to lymphoma cells. In order to delineate whether SU-DHL-2/4 bind with Tandab(IL-6/CD20) protein, UCMSCs-Tandab(IL-6/CD20) were cultured in the inserts (5 × 10^3^ cells) and SU-DHL-2/4 (1 × 10^4^ cells) were seeded in the lower chamber with a 14-mm cell climbing slice coated with polylysine. After co-cultured for 72 h, the inserts were removed and the lower chamber with cell climbing slice was washed, fixed and then co-incubated with rabbit anti-human His-Tag and mouse anti-human CD20 (302,306, BioLegend, The USA) antibodies for 1 h; cells were then incubated with Alexa Fluor® 594 Conjugated goat anti-rabbit and Alexa Fluor® 488 Conjugated goat anti-mouse IgG (H + L) secondary antibodies (#4408, Cell Signaling Technology, The USA). After washing by cold PBS, the nuclei were stained with DAPI staining solution for 3 min. The images were captured by using a single photon confocal microscopy (Ti-E A1, Nikon, Japan).

SU-DHL-2 or SU-DHL-4 (1 × 10^6^ cells) were co-cultured with UCMSCs-Tandab(IL-6/CD20) (2 × 10^5^ cells) in a 6-well plate transwell system for 24 h in a humidified incubator at 37℃. After collected and washed twice, the SU-DHL-2 or SU-DHL-4 were incubated with rabbit anti-human His-Tag and goat anti-rabbit IgG H&L (FITC) (ab6717, Abcam, The USA) antibodies for 1 h and analyzed in a flow cytometer (C6, BD Biosciences, The USA).

In competitive binding assay, lymphoma cells were co-cultured with UCMSCs-Tandab(IL-6/CD20) (2 × 10^5^ cells) in a 6-well transwell plate for 24 h in a humidified incubator. Subsequently, the cells were incubated with PE anti-human CD20 antibody (302,306, BioLegend, The USA) for 1 h and analyzed by flow cytometry. The expressions were presented in a histogram graph by using the FlowJo software (10.5.3, The USA).

### Viability and proliferation assay

Cell viability and proliferation were assessed by CCK-8 (C0043, Beyotime, China) and 5-ethynyl-2’-deoxyuridine (EdU) (C0071S, Beyotime, China) labeling analyses. Briefly, in the CCK-8 assay, SU-DHL-2 or SU-DHL-4 (2 × 10^3^ cells/well) were cultured with or without PBMCs (2 × 10^3^ cells/well) in 96-well plates. The supernatants of UCMSCs-Tandab(IL-6/CD20), rituximab (MB2749, Meilunbio, China)(10 ng/ml, 100 ng/ml, 1000 ng/ml), tocilizumab (MB2751, Meilunbio, China)(10 ng/ml), and rituximab + tocilizumab (5 and 5 ng/ml) were added to the culture medium, respectively. After cultured for 24, 48, 72, and 96 h, cells were analyzed by using CCK-8 assay according to manufacturer's protocol; the absorbances were read at 450 nm in a Microplate Reader (Cytation 5, Bio-Tek, The USA).

In order to assess the efficacy of PBMCs, SU-DHL-2 or SU-DHL-4 (4000 cells) and PBMCs were seeded in 96-well plate at different effector to target (E/T) cell ratios (0.1:1, 1:1, 10:1) with or without the supernatants of UCMSCs-Tandab(IL-6/CD20) treated. After co-cultured for 24, 48, 72, and 96 h, cells were analyzed by using CCK-8 assay and the absorbances were read at 450 nm in a Microplate Reader (Cytation 5, Bio-Tek, The USA).

In the EdU assay, SU-DHL-4 cells were treated with twelve groups including the UCMSCs-Tandab(IL-6/CD20), UCMSCs-Vector, UCMSCs, rituximab (10 ng/ml), tocilizumab (10 ng/ml), and rituximab + tocilizumab (5 and 5 ng/ml), respectively, with or without treating with PBMCs. In groups untreated with PBMCs, cells were cultured in a 24-well plate with transwell inserts. UCMSCs-Tandab(IL-6/CD20), UCMSCs-Vector, and UCMSCs (2 × 10^4^ cells/well) were seeded in the inserts and SU-DHL-4 cells were cultured in the lower chamber with a climbing slice coated with polylysine. After co-cultured for 72 h, the inserts were removed. The SU-DHL-4 cells were exposed to 10 μM EdU for 2 h, cells were fixed with 4% paraformaldehyde and then stained with Hoechst 33,342 for nuclear stain. EdU-positive cells were calculated in five random fields under a single photon confocal microscopy (Ti-E A1, Nikon, Japan). In the PBMC-treated groups, SU-DHL-4 cells were stained with 2 nM PKH26 (MINI26-1KT, Sigma-Aldrich, The USA) in advance according to the manufacturer’s instruction. Then, SU-DHL-4 (2 × 10^4^ cells/well) and PBMCs (2 × 10^4^ cells/well) were seeded in 24-well plates. The remaining steps were the same as mentioned above.

### Statistical analysis

All parameters were presented as mean ± SD percentage positivity. Statistical analyses were performed by using GraphPad Prism (8.0.1, GraphPad Software, The USA). One-way or two-way ANOVA was used to obtain the significance of the difference. *P* values of less than 0.05 were considered significant.

## Results

### The construction of UCMSCs-Tandab(IL-6/CD20)

**Fig. 1:**
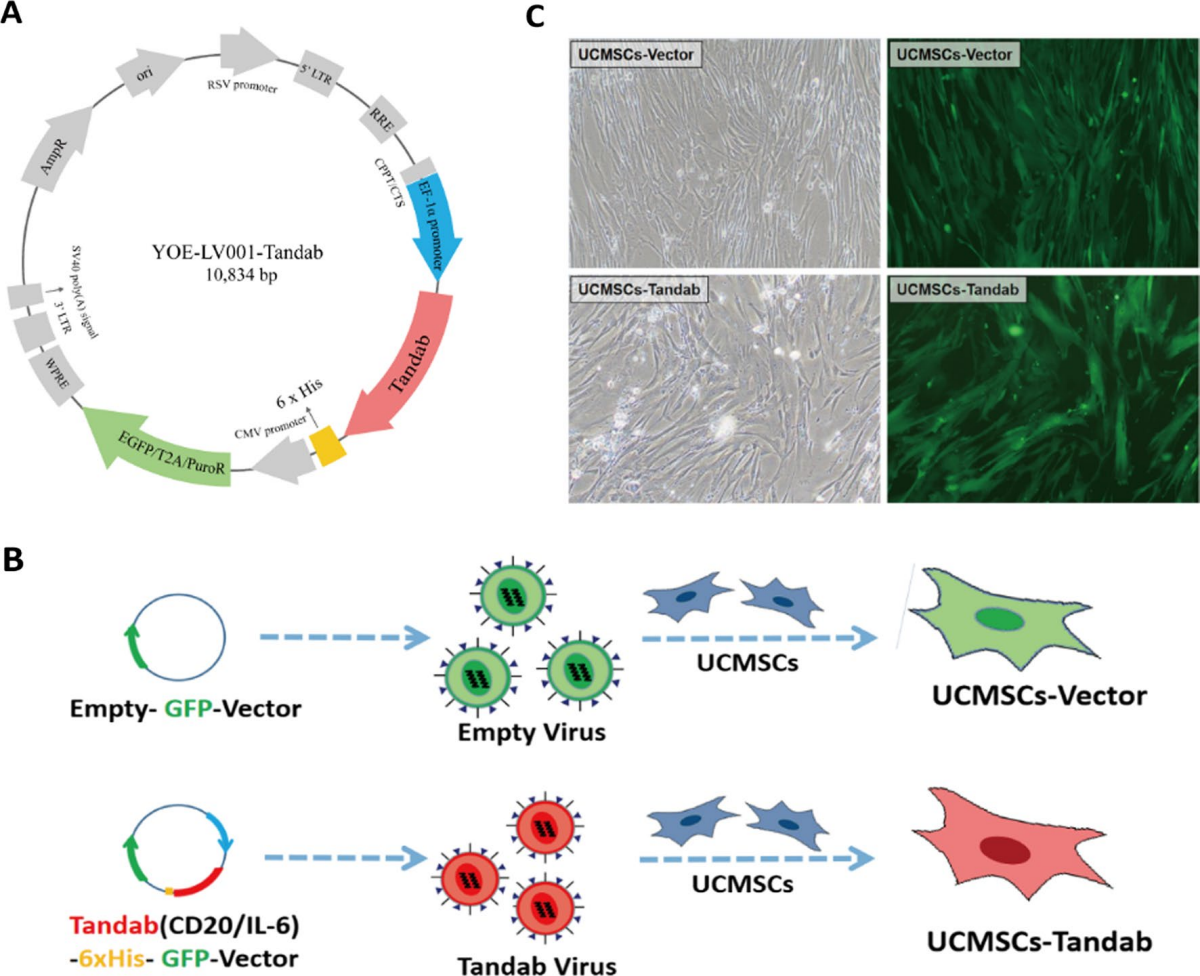
Construction of UCMSCs-Tandab(IL-6/CD20). **A** Schematic diagram of the Tandab recombinant vector (Tandab(CD20/IL-6)-6 × His-GFP vector). **B** Schematic representation to produce UCMSCs-Tandab(IL-6/CD20) and UCMSCs-Vector cell lines. With a lentivirus package, the Empty-GFP vector and Tandab(CD20/IL-6)-6 × His-GFP vector transfected HEK293T cells to collect empty and Tandab virus. Then, the empty and Tandab virus infected UCMSCs to produce UCMSCs-Vector and UCMSCs-Tandab(IL-6/CD20), respectively. **C** The morphology and fluorescence image of UCMSCs transfected with Tandab virus or empty virus under an inverted fluorescence microscope (Scale bar: 100 μm). Abbreviations: Tandab: Tandem diabody, GFP: Green fluorescent protein, 6 × His: Hexa-histidine tag

We have designed the gene sequence of tandem diabody (IL-6/CD20) (Tandab(IL-6/CD20)). The full length of the objective gene sequence contains 1506 base pair, and the gene makeup was as follows: CD33SP-VLCD20Ab-GGGGS-VHCD20Ab-3 × GGGGS-VLIL-6Ab-GGGGS-VHIL-6Ab-6 × His. Guangzhou Yuanjing Biotechnology company (Ubigene, China) was entrusted to synthesize and construct the experimental vector encoding Tandab(IL-6/CD20) gene (Tandab(CD20/IL-6)-6 × His-GFP vector) and the empty vector with GFP cDNA (Empty-GFP vector) (Fig. 1A). Tandab gene was amplified and subcloned into the lentivirus vector YOE-LV001 to produce lentivirus vector YOE-LV001-Tandab (Tandab(CD20/IL-6)-6 × His-GFP vector). The Tandab virus was produced from the transient transfection of the HEK293T cells with Tandab(CD20/IL-6)-6 × His-GFP vector together with pCMV-VSVG envelope vector and psPAX2 packaging vector. After transfected HEK293T cells, a mass of empty and fusion Tandab viruses were obtained. The gene sequence of the Tandab virus was verified correctly by sequence analysis.

Tandab virus and empty virus were used to infect UCMSCs to produce UCMSCs-Tandab(IL-6/CD20) and UCMSCs-Vector at a MOI of 10, respectively. UCMSCs-Vector and UCMSCs were defined as negative control groups in our following experiments. After screened with puromycin, the purified UCMSCs-Tandab(IL-6/CD20) and UCMSCs-Vector were obtained (Fig. 1B). These modified cells were cultured for 4 days, and the morphology of UCMSCs-Tandab(IL-6/CD20) and UCMSCs-Vector was observed under a microscope, showing spindle-shaped and growing adherently to culture plates. Both of modified cells show spontaneous green fluorescence under an inverted fluorescence microscope, suggesting that the cells were successfully infected (Fig. 1C).

### Characterization of UCMSCs via flow cytometry

The expression of cell surface markers was analyzed in UCMSCs (Additional file 1: Fig S1), showing that UCMSCs-Tandab(IL-6/CD20), UCMSCs-Vector and UCMSCs were positive for mesenchymal markers CD90 (93.1%, 99%, 99.6%) and CD105 (90.3%, 93.5%, 95.2%), but negative for hematopoietic markers CD34 (0.14%, 0.78%, 0.9%).

### Vitality and migration abilities of UCMSCs-Tandab(IL-6/CD20)

**Fig. 2:**
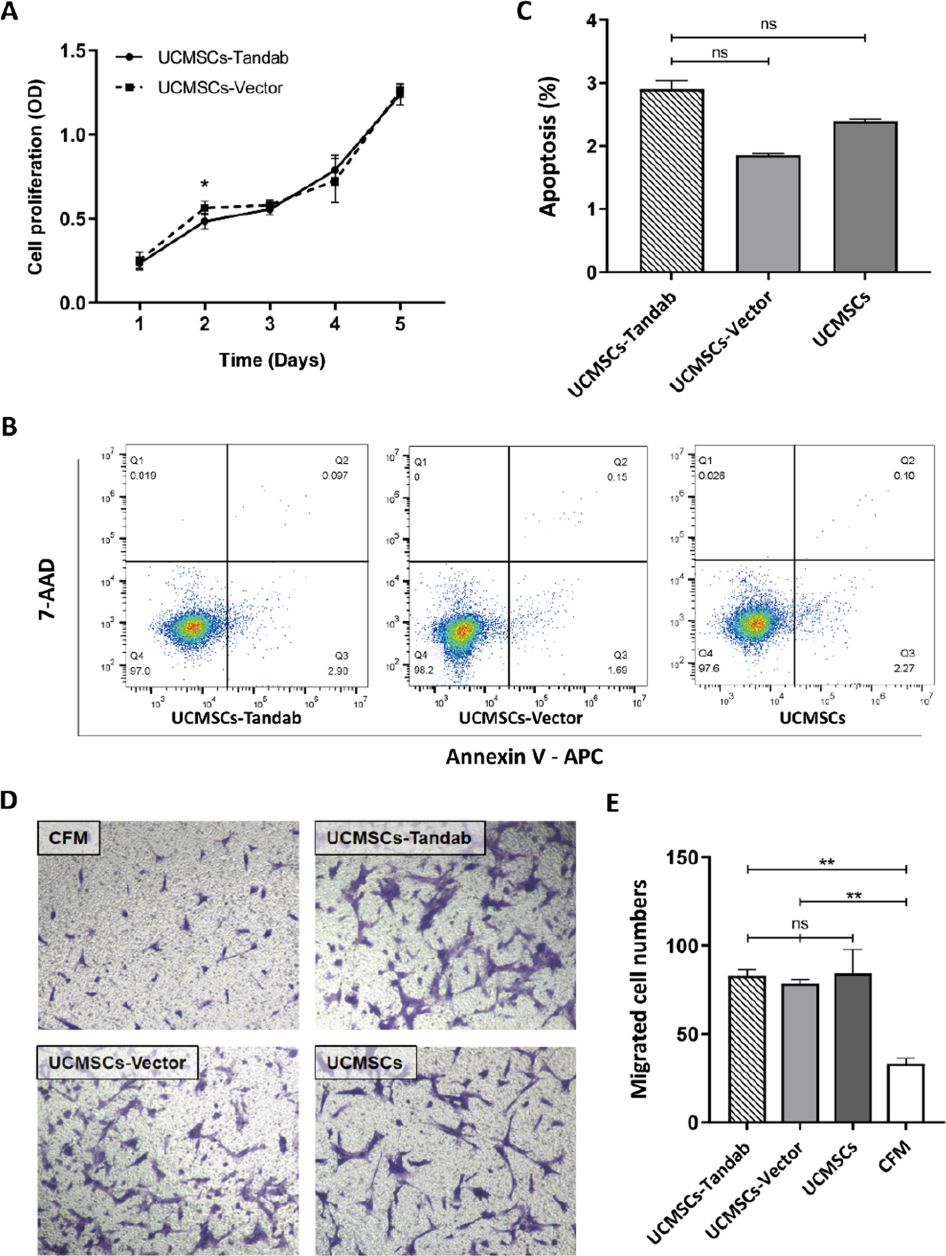
Vitality, apoptosis and homing abilities of modified UCMSCs. **A** Cell viability of UCMSCs-Tandab(IL-6/CD20) and UCMSCs-Vector by CCK-8 assay. *n* = 5 for each group. **B** Apoptosis assay of UCMSCs-Tandab(IL-6/CD20), UCMSCs-Vector and UCMSCs by flow cytometry analysis. The histogram of apoptosis is shown in (**C**). (**D**) The migration ability of all three UCMSCs lines by transwell migration assay. Supernatants of SU-DHL-4 were used to trigger cells (UCMSCs-Tandab(IL-6/CD20), UCMSCs-Vector and UCMSCs) migration, and the CFM group served as a negative control. The numbers of migrated cells were statistically analyzed in (**E**). *n* = 3 for each group. ns: not significant, *p < 0.05, **p < 0.01. Abbreviations: CFM: Cell-free medium

To investigate the effect of Tandab lentivirus in the survival of UCMSCs, we evaluated the cell vitality and apoptosis in UCMSCs-Tandab(IL-6/CD20). CCK-8 and apoptosis assay were utilized to show the proliferation of UCMSCs-Tandab(IL-6/CD20) that makes no difference with UCMSCs-Vector, suggesting that Tandab lentivirus had no influence on modified UCMSCs (Fig. 2A–C). In order to investigate the migration ability of UCMSCs-Tandab(IL-6/CD20), wound healing scratch assay was used. The results demonstrated no significant influence when compared with the UCMSCs-Vector and UCMSCs groups after cultured for 12 h and 24 h (Additional file 1: Fig S2).

Transwell migration assay was performed in an attempt to investigate the migration capacity of modified UCMSCs to SU-DHL-4 cells. Cell-free group was defined as a negative control of chemotaxis, while UCMSCs-Tandab(IL-6/CD20), UCMSCs-Vector and UCMSCs were used as positive control of migrating cells. Compared with the cell-free group, SU-DHL-4 could stimulate the migration of MSCs, whereas the migration capacity of MSCs was not affected by infection of lentivirus (Fig. 2D–E).

### Expression of Tandab fusion protein

**Fig. 3:**
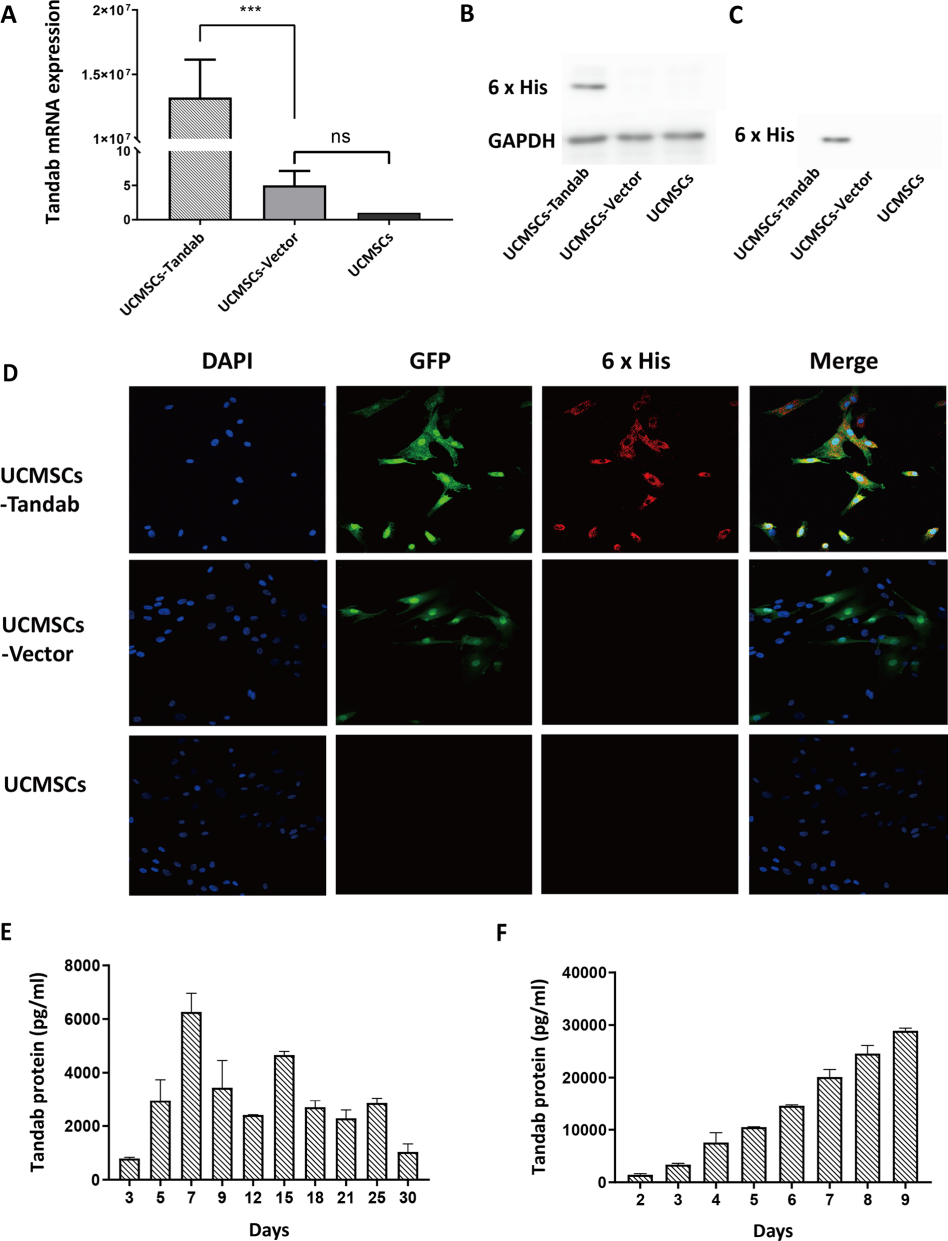
The expression of Tandab protein. (**A**) The relative expression of Tandab mRNA in UCMSCs-Tandab(IL-6/CD20), UCMSCs-Vector and UCMSCs analyzed by qRT-PCR. (**B-C**) The relative expression of Tandab protein in the cell lysis (**B**) and the culture supernatant (**C**) of UCMSCs-Tandab(IL-6/CD20) by western blot. (**D**) Representative immunofluorescence staining of UCMSCs-Tandab(IL-6/CD20), UCMSCs-Vector and UCMSCs. Green and red represent spontaneous fluorescence and 6 × His Tag, respectively. (**E–F**) After collecting the culture supernatants of UCMSCs-Tandab(IL-6/CD20), the Tandab protein level was measured by ELISA. *n* = 3 for each group. ns: not significant, ***p < 0.001

To detect whether UCMSCs-Tandab(IL-6/CD20) express the inserted Tandab gene, total RNA was isolated, and subsequently, quantitative real-time PCR was used to detect the expression level of Tandab mRNA in all three UCMSCs lines. The results showed a high expression of Tandab mRNA in UCMSCs-Tandab(IL-6/CD20) (Fig. 3A). We found that Tandab protein tagged with 6 × His Tag was detected both in the cell lysis and in the culture supernatant of UCMSCs-Tandab(IL-6/CD20) by western blot (Fig. 3B–C). Furthermore, we evaluated the fluorescence image under a confocal microscope by staining 6 × His Tag antibody and DAPI solution in the three UCMSC cell lines. UCMSCs-Tandab(IL-6/CD20) and UCMSCs-Vector showed spontaneous green fluorescence and only UCMSCs-Tandab(IL-6/CD20) could be stained by 6 × His Tag antibody showing red fluorescence (Fig. 3D). The UCMSCs-Tandab(IL-6/CD20) were cultured in a 6-well plate, and the secretion of Tandab protein was detected by collecting and changing the culture medium every day. The Tandab (IL-6/CD20) released in the supernatants in culture of UCMSCs-Tandab was detected by ELISA. UCMSCs-Tandab(IL-6/CD20) secreting level was continuously increasing with the culture days and reached the peak concentration of 6273 ± 487 pg/ml at day 7 (Fig. 3E). The total secretion of Tandab protein reached 28,927 ± 406 pg/ml at day 9 (Fig. 3F) and was detectable even at day 30.

### Verification of Tandab bounding to CD20-positive SU-DHL-2/4 cells

**Fig. 4:**
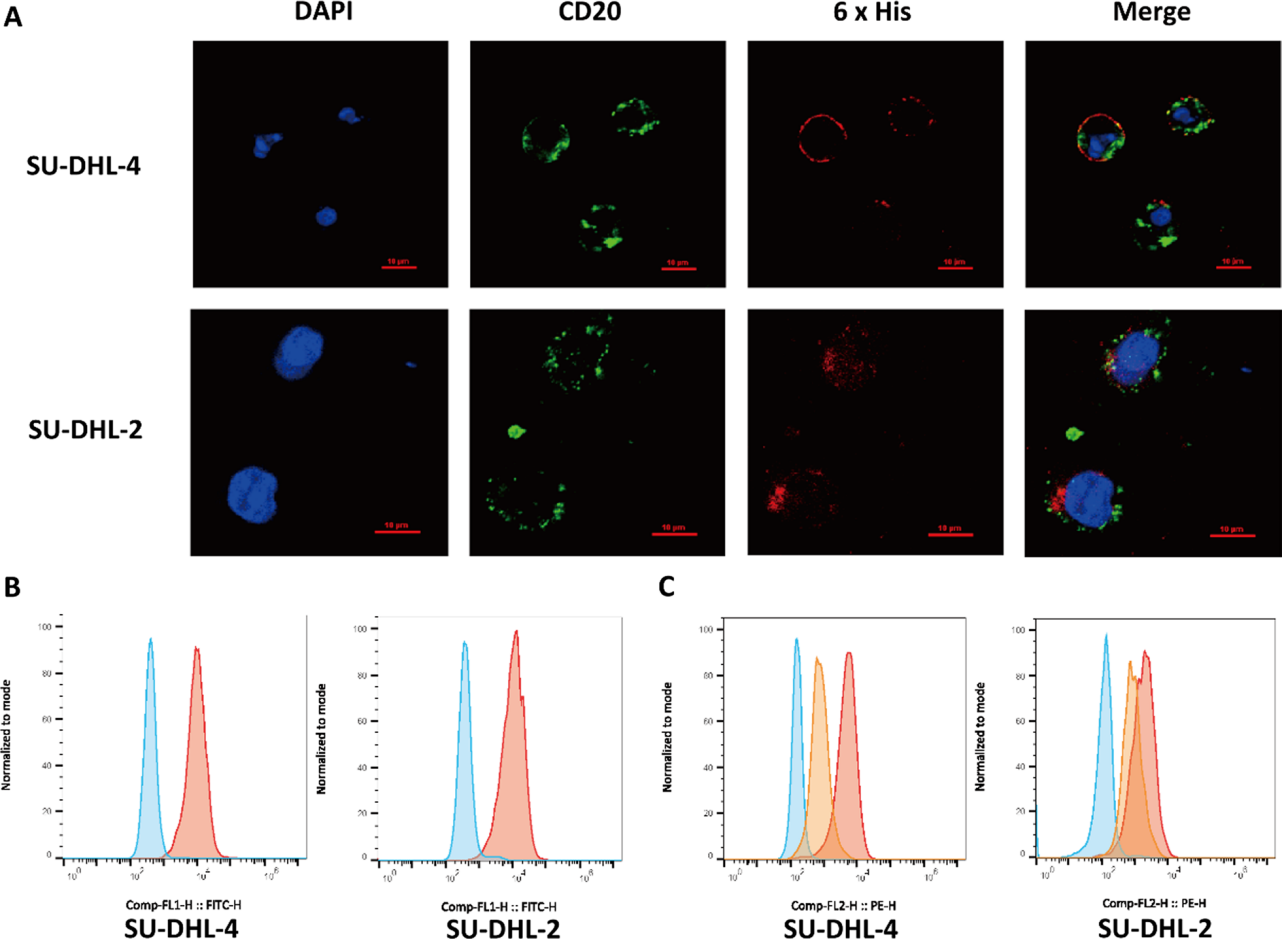
Tandab bind to the CD20-positive SU-DHL-2/4 cells. **A** The fluorescence image of Tandab bound to SU-DHL-2 and SU-DHL-4 cells. Green and red represent CD20 and 6 × His Tag, respectively. **B** Flow cytometric analysis of Tandab bound to SU-DHL-2/4 cells. Blue: negative control, red: experimental groups treated with UCMSCs-Tandab(IL-6/CD20). **C** The competitive binding assay with anti-human CD20 monoclonal antibody. Blue: negative control, red: Tandab(IL-6/CD20) + anti-human CD20 monoclonal antibody, orange: anti-human CD20 monoclonal antibody alone

UCMSCs-Tandab(IL-6/CD20) were cultured in the transwell inserts and SU-DHL-2 or SU-DHL-4 cells were seeded in the lower chamber. After co-cultured for 72 h and stained by primary and secondary antibodies, the results showed that SU-DHL-2 and SU-DHL-4 cells express CD20 antigen (green) and were bound by Tandab protein (red) which contained IL-6 and CD20 fusion antibody (Fig. 4A). The binding specificity of Tandab targeting CD20-positive B cell was also detected by flow cytometry analysis. The results suggested that both SU-DHL-2 and SU-DHL-4 could bind to Tandab protein (red), and the isotype group was used as negative control (blue) (Fig. 4B). In competitive binding assay, lymphoma cells in experimental group were co-cultured with UCMSCs-Tandab(IL-6/CD20) in a transwell system for 24 h and then the cells were incubated with anti-human CD20 antibody and analyzed by flow cytometry. Compared to the group untreated with UCMSCs-Tandab(IL-6/CD20) (red), the curve shifted to the left in experimental group (orange), indicating that the positive rate of CD20 decreased. Therefore, after co-cultured with lymphoma cells, Tandab (IL-6/CD20) protein competitively inhibited the binding of human CD20 monoclonal antibody to the CD20 antigen. These results indicated that Tandab protein containing CD20 antibody could bind the CD20 antigen on B-lymphoma cells (Fig. 4C).

### UCMSCs-Tandab(IL-6/CD20) inhibited SU-DHL-2/4 proliferation

**Fig. 5:**
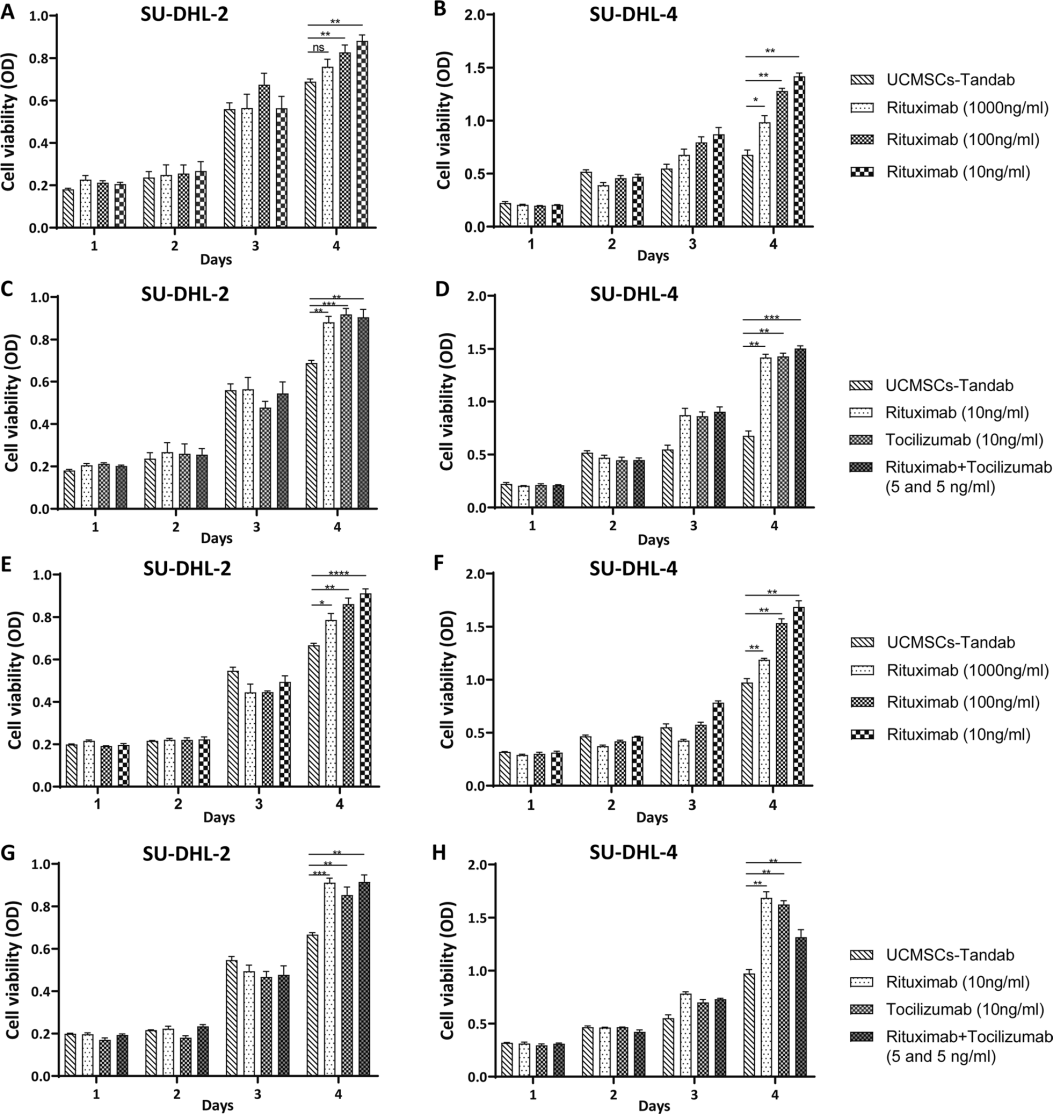
UCMSCs-Tandab(IL-6/CD20) inhibited the proliferation of SU-DHL-2/4. (**A-D**) CCK-8 assay for evaluating the cell viability of SU-DHL-2 (**A** and **C**) or SU-DHL-4 (**B** and **D**) after different treatments (the supernatants of UCMSCs-Tandab(IL-6/CD20), Rituximab (1000 ng/ml, 100 ng/ml, 10 ng/ml), Tocilizumab(10 ng/ml) and mixture of Rituximab and Tocilizumab (5 and 5 ng/ml)) without the presence of PBMCs. (**E–H**) CCK-8 assay for evaluating the viability of SU-DHL-2 (**E** and **G**) or SU-DHL-4 (**F** and **H**) after different treatments (the supernatants of UCMSCs-Tandab(IL-6/CD20), Rituximab(1000 ng/ml, 100 ng/ml, 10 ng/ml), Tocilizumab(10 ng/ml) and mixture of Rituximab and Tocilizumab (5 and 5 ng/ml)) with the presence of PBMCs. *n* = 6 for each group. ns: not significant, *p < 0.05, **p < 0.01, ***p < 0.001

To identify the cell viability of SU-DHL-2 and SU-DHL-4 after treated with the supernatants of UCMSCs-Tandab(IL-6/CD20) or UCMSCs-Tandab(IL-6/CD20), we collected the supernatant of UCMSCs-Tandab(IL-6/CD20) after cell cultured for 5 days, detected the Tandab concentration by using a 6 × His Tag ELISA kit and standardized to 10 ng/ml. CCK-8 assay was used to compare the therapeutic effect of the supernatants of UCMSCs-Tandab(IL-6/CD20) against lymphoma cells with rituximab, tocilizumab and rituximab + tocilizumab in vitro. In the absence of PBMC co-incubation, the inhibition effect of the UCMSCs-Tandab(IL-6/CD20) groups was superior to the rituximab-treated groups (10 ng/ml, 100 ng/ml) (Fig. 5A–B). The cell viability of SU-DHL-4, rather than SU-DHL-2, in UCMSCs-Tandab(IL-6/CD20) groups was lower than rituximab groups (1000 ng/ml) (Fig. 5A–B). In addition, the cell viability of SU-DHL-2 and SU-DHL-4 in UCMSCs-Tandab(IL-6/CD20) group was lower than tocilizumab, rituximab, and rituximab and tocilizumab combined treatment groups (Fig. 5C–D). Previous researches indicated that rituximab exerts its effects by inducing apoptosis, antibody-dependent cell cytotoxicity (ADCC) and complement-dependent cellular cytotoxicity (CDCC) mechanisms. We extracted PBMCs by density gradient centrifugation from healthy donors. SU-DHL-2 or SU-DHL-4 cells were co-cultured with PBMCs in different treatments. The results were in line with the groups without PBMCs; the cell viability of SU-DHL-2 (Fig. 5E and 5G) and SU-DHL-4 (Fig. 5F and 5H) in UCMSCs-Tandab(IL-6/CD20) groups was lower than other groups.

**Fig. 6:**
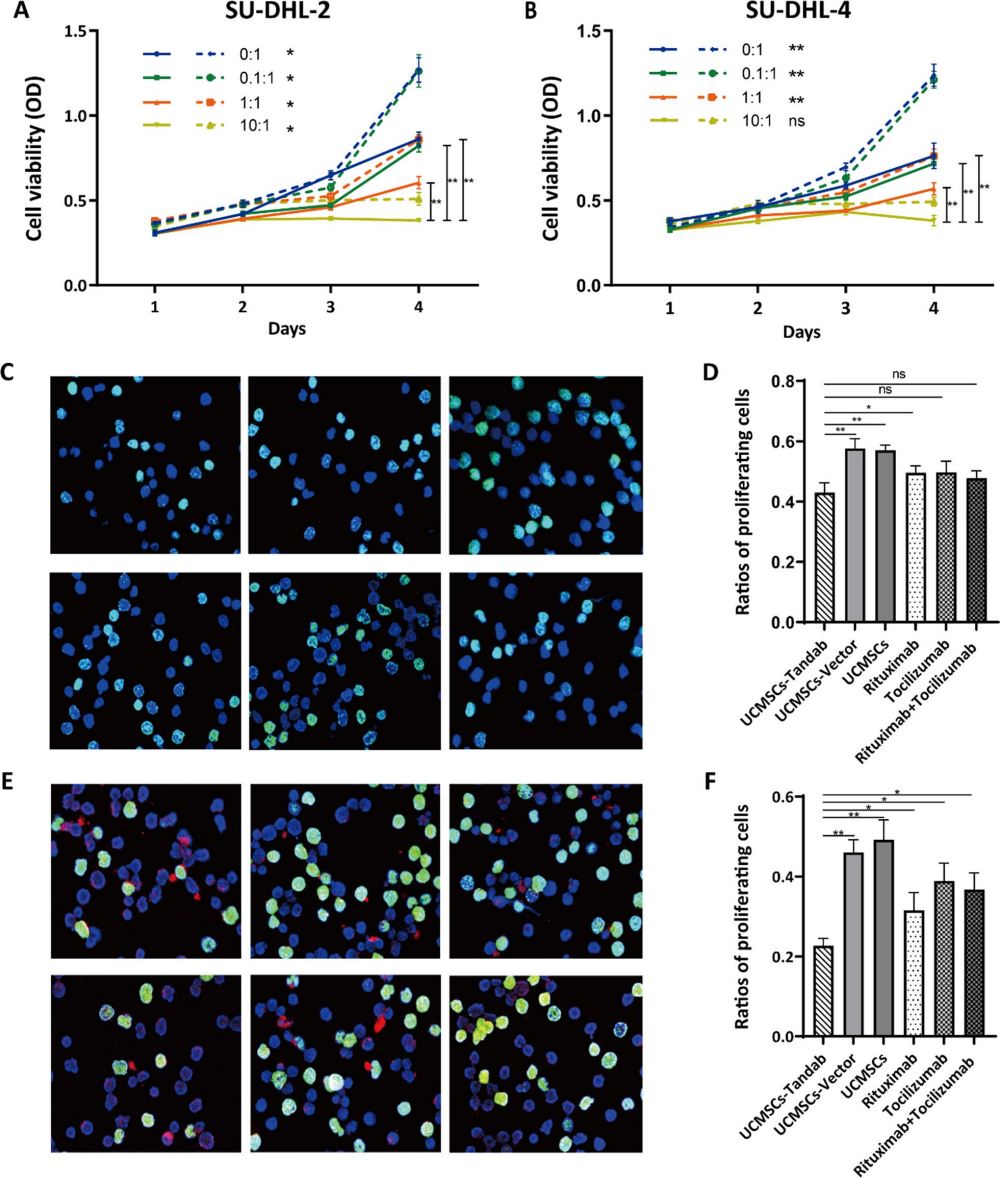
UCMSCs-Tandab(IL-6/CD20) inhibited the proliferation of SU-DHL-2/4. (**A-B**) CCK-8 assay to detect the toxicity effective of different E/T values (0:1, 0.1:1, 1:1, 10:1) on SU-DHL-2 (A) or SU-DHL-4 (**B**) cells. The solid lines indicate the groups treated with the supernatants of UCMSCs-Tandab(IL-6/CD20). *n* = 6 for each group. (**C-F**) EDU assay to evaluate the cell viability of SU-DHL-2 or SU-DHL-4 after different treatments (UCMSCs-Tandab(IL-6/CD20), UCMSCs-Vector, UCMSCs, Rituximab, Tocilizumab, mixture of Rituximab and Tocilizumab) with (**E–F**) or without (**C-D**) the presence of PBMCs and analytical results of positively stained EDU-positive cells to detect the percentage of proliferating cells after cell cultured for 72 h in (**D**) and (**F**). *n* = 5 for each group. ns: not significant, *p < 0.05, **p < 0.01

The toxicity experiment with different effector-to-target (E/T) values (0:1, 0.1:1, 1:1, 10:1) was used to assess the potential effect of PBMCs in the co-culture of both SU-DHL-2/4 and UCMSCs-Tandab (IL-6/CD20). The results showed that the proliferation of either SU-DHL-2 (Fig. 6A) or SU-DHL-4 cells (Fig. 6B) was inhibited with the increase in E/T values. Furthermore, the inhibition effect of group treated with UCMSCs-Tandab(IL-6/CD20) was significantly greater than control groups. EDU assay was used to strengthen the data; the ratio of proliferating cells showed that the cell proliferation of lymphoma cells was lower in UCMSCs-Tandab(IL-6/CD20) groups compared with other groups (UCMSCs-Vector, UCMSCs, rituximab, tocilizumab and combined treatment of rituximab and tocilizumab). The data showed that there was no significant difference between UCMSCs-Tandab(IL-6/CD20) group and rituximab-treated group (*P* > 0.05) when PBMCs are absent (Fig. 6C–D). When co-cultured with PBMCs, the ratio of proliferating cells in UCMSCs-Tandab (IL-6/CD20) treatment group was 22.66%, while in rituximab group was 31.54% (*P* < 0.05) (Fig. 6E–F).

## Discussion

In this study, we are the first to establish a Tandem diabody and construct UCMSCs-Tandab (IL-6/CD20) and investigated the therapeutic effects of gene-modified MSCs with Tandab (IL-6/CD20) for DLBCL. The results demonstrated that UCMSCs-Tandab (IL-6/CD20) targeting CD20-positive DLBCL cells, evidenced by bounding to CD20-positive SU-DHL-2/4 cells, inhibit the proliferation of tumor cells by downregulating IL-6-related signaling pathway.

A combination treatment of rituximab and CHOP has been introduced as the first-line chemotherapy regimen for DLBCL [3]. Rituximab, an anti-CD20 chimeric antibody that specifically targets the CD20-positive lymphoma cells, has anti-tumor effects by inducing apoptosis, CDCC, ADCC, and cross-priming mechanisms [26]. Theoretically, antibody-based therapy could eliminate all tumor cells; however, it is reported that 4–12% of patients who received the rituximab-based therapy experienced primarily refractory and 17–40% relapse after complete remission (CR) or partial remission (PR). Moreover, 12–60% of these patients experience tumor recurrence after the second- or third-line therapy [2–4, 27]. The resistance mechanisms of rituximab remain elusive, which might be associated with tumor microenvironment (TME), host immunologic factors, pharmacokinetics, downregulation or loss of CD20 [26, 28]. Accumulating evidence indicated that TME is related to anti-tumor drug resistance due to the cytokines secreted by TME cells, such as lymphocytes, macrophages, endothelial cells and MSCs. These cytokines (IL-1, IL-6, TNF, IL-17, etc.) have been proved to promote tumor cell proliferation, angiogenesis and metastasis [10, 29, 30]. In our previous study, we have found that naive Karpas1106 cells express detectable IL-6, which could be upregulated after the irradiation treatment. IL-6 derived from irradiated Karpas1106 cells could induce regulatory T cells (Tregs) to express IL-17, which inhibited the expression of tumor suppressor p53 protein and induced the production of drug resistance [31]. Our retrospective analysis demonstrated that rituximab-based therapy increased the expression of IL-6 in patients with DLBCL [32]; furthermore, the cell culture study demonstrated that rituximab significantly increased the expression of IL-6 in SU-DHL-4 cells both in protein and in mRNA levels with a concentration-dependent manner [32]. IL-6 increased IL-17A level by inducing the differentiation of Th17 and Treg cells, which could be markedly promoted by exogenous IL-6 and inhibited by anti- IL-6 antibody [32]. Other studies were congruent with our findings that IL-6 serves as a negative prognostic factor for DLBCL, as well as a promising therapeutic target [8–10]. Previous study demonstrated that DLBCL has pro-tumor mechanisms by expressing IL-6/IL-6R and inhibiting negative regulator, eventually promoting the constitutive activation of IL-6/IL-6R axis [33]. Therefore, the progress of DLBCL is plausibly associated with the TME, while IL-6 secreted by TME-related cells plays a pivotal role in tumor growth. Anti-IL-6 antibody could abrogate the IL-6/IL-6R axis and promote the apoptosis of lymphoma cells [7, 11, 31, 32]. The neutralization of IL-6 with anti-IL-6 antibodies and the abrogation of IL-6/IL-6R signaling pathway could relieve rituximab resistance and prevent the growth of DLBCL. To this end, we have constructed a cellular delivery vehicle by genetically engineering, which would produce antibodies that neutralize pro-tumor factors and target tumor surface antigens in the TME, and exert anti-tumor effect in tumor loci.

Cell-based therapy has emerged as a novel approach to treat malignancies. In the current study, we have successfully constructed a Tandab lentivirus and infected UCMSCs to produce UCMSCs-Tandab(IL-6/CD20). UCMSCs could maintain their properties after genetic modification, including their morphology and immunophenotype. In accordance with the proliferation assay and Annexin V results, it was well identified that the cell viability and proliferation were not impaired after modified. Previous evidence indicated that MSCs were easy to be genetically modified and stay stable [17, 34]. UCMSCs-Tandab(IL-6/CD20) secreting level was continuously increasing with the culture days, reached the peak concentration of 6273 ± 487 pg/ml at day 7, and was detectable even at day 30, suggesting that the secretion of the Tandab protein was continuous and stable. The result was in accordance with other studies that reported the highest level at 6–9 days ranging from 1500 to 8247 pg/ml and could constantly increase for 15 to 30 days after transduction [23, 25]. Previous evidence has indicated that the modified MSCs could serve as an excellent local agent delivery vehicle for its innate homing ability [35–37]. The modified MSCs in conjunction with targeted drugs have been introduced in clinical practice: engineered UCMSCs produced TRAIL for intracranial glioma [38], and MSCs loaded with oncolytic reovirus ReoT3D for colorectal cancer [39]. In our study, UCMSCs-Tandab(IL-6/CD20) could migrate toward cell-free medium; the migration could be promoted when co-cultured with SU-DHL-4 cells. These results indicated the migratory ability of MSCs did not weaken after lentivirus infection. Another research proved that the transduced MSCs labeled with luciferase could selectively migrate to the tumor loci 24 h after intravenous injection [25]. The modified MSCs could survive in tumor region for more than 3 weeks [40]. The magnitude of scFv EGFRvIII expressed by MSCs was significantly greater than MSCs control group in tumor region at day 5 and day 15 [41], suggesting that the modified MSCs expressing antibodies against tumor surface antigens might increase the retention in vivo.

In preclinical studies, the most common inserted gene fragments are cytokines, pro-apoptotic proteins, oncolytic proteins and antibodies targeting tumor cells [38, 39, 42]. The previous clinical trial results showed that genetically modified MSCs were safe and effective for treating patients with lymphoma [17]. MSCs expressing EGFRvIII light- and heavy-chain fragments have been shown effective in the treatment of mouse glioblastoma [41]. Further studies have demonstrated that two different antibody fragments could be transfected into MSCs by linker fragments, which secreted antibodies and expressed more targeted agents [43]. It has been proved that CD3 and CD19 bispecific antibodies expressed by MSCs could be used in the treatment of CD19-positive Raji lymphoma [25]. Another study showed that MSCs, which expressed anti-PSA antibody and BH3-interacting domain death agonist (BID, a member of Bcl-2 protein family) fusion protein, could be used to treat prostate cancer [23]. All these existing studies have shown that MSCs, as the vehicle of antibodies, have good affinity to the tumor and tumor-killing activity. On the basis of MSCs homing toward tumor region and the combination of CD20 antibody to CD20-positive B cell lymphocytes, UCMSCs-Tandab(IL-6/CD20) could effectively inhibit the viability of lymphoma cells in the ex vivo experiment. The proliferation inhibiting rate of SU-DHL-2 and SU-DHL-4 in UCMSCs-Tandab(IL-6/CD20) treatment groups was significantly higher than other treatment groups (UCMSCs-Vector, UCMSCs, rituximab, tocilizumab, and rituximab + tocilizumab). Co-cultured with PBMCs, the ratio of proliferating cells in UCMSCs-Tandab(IL-6/CD20) treatment group decreased from 42.98% to 22.66%, while in rituximab group reduced from 49.52 to 31.54%, indicating that UCMSCs-Tandab(IL-6/CD20) were effective in inhibiting lymphoma cells, while PBMCs enhance the curative efficacy, which has been proved in the study of modified MSCs-Tandab(CD3/CD19) on B cell lymphoma [35]. Furthermore, our results showed that the cytotoxicity efficacy was in dose-dependent manner and could be enhanced with the increase in E/T ratio. Modified MSCs possess the tropism toward tumor, express the target protein locally, increase the concentration of anti-tumor drugs in the tumor loci, as well as conduct a long-term effect on inhibiting tumor growth.

In sum, the Tandab(IL-6/CD20) protein could target CD20-positive DLBCL cells, as well as inhibit IL-6-related signaling pathway and eliminate the promoting effect of IL-6 on lymphoma cells. However, tumor-bearing animal experiments and clinical treatment should be carry out in future to prove its anti-tumor effect. Despite numerous studies, the underlying molecular and cellular mechanisms have yet to be fully elucidated.

## Conclusion

A novel UCMSCs-Tandab(IL-6/CD20) could be used to bind with the CD20-positive lymphoma cells and pro-tumor cytokines IL-6, target and kill tumor cells. Genetically modified UCMSCs expressing tandem diabody (IL-6/CD20) might be a potential strategy in the treatment of DLBCL.

## Supplementary Information

**Additional file 1: Fig. S1 **The immunophenotype of modified UCMSCs. **Fig. S2 **The migration ability of modified UCMSCs

## Data Availability

All data generated or analyzed during this study are included in this published article and its supplementary information files.

## Abbreviations

DLBCL: Diffuse large B cell lymphoma
R/R: Relapse or refractory
NHL: Non-Hodgkin’s lymphoma
ADCC: Antibody-dependent cell cytotoxicity
CDCC: Complement-dependent cell cytotoxicity
ABC DLBCL: Activated B cell-like DLBCL
GCB DLBCL: Germinal center B cell-like DLBCL
CR: Complete remission
PR: Partial remission
TME: Tumor microenvironment
