# Supplementary material for: Construction of tandem diabody (IL-6/CD20)-secreting human umbilical cord mesenchymal stem cells and its experimental treatment on diffuse large B cell lymphoma
Source: Stem Cell Res Ther. 2022 Sep 14;13:473. doi: 10.1186/s13287-022-03169-4 (PMC9476312; doi:10.1186/s13287-022-03169-4)
Supplement: Supplementary file 1 — Additional file 1: Fig. S1 The immunophenotype of modified UCMSCs. Fig. S2 The migration ability of modified UCMSCs [file 13287_2022_3169_MOESM1_ESM.docx]

**Supplementary Material**


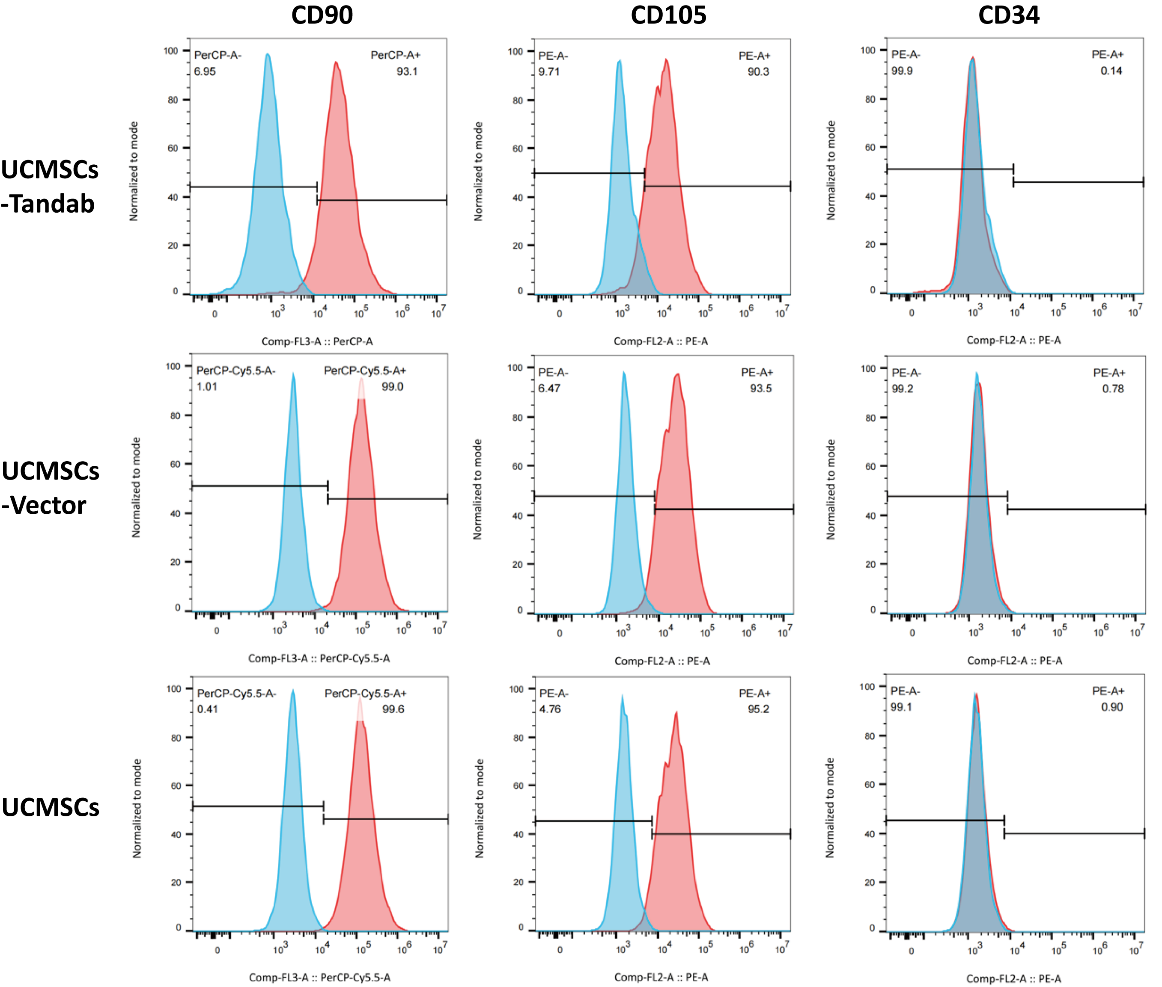


**Figure S1. The immunophenotype of modified UCMSCs**

The expression of CD34, CD90, CD105 on UCMSCs-Tandab(IL-6/CD20), UCMSCs-Vector and UCMSCs by flow cytometry analysis. UCMSCs cells lines (red) and isotype controls (blue) were presented in histogram graph.


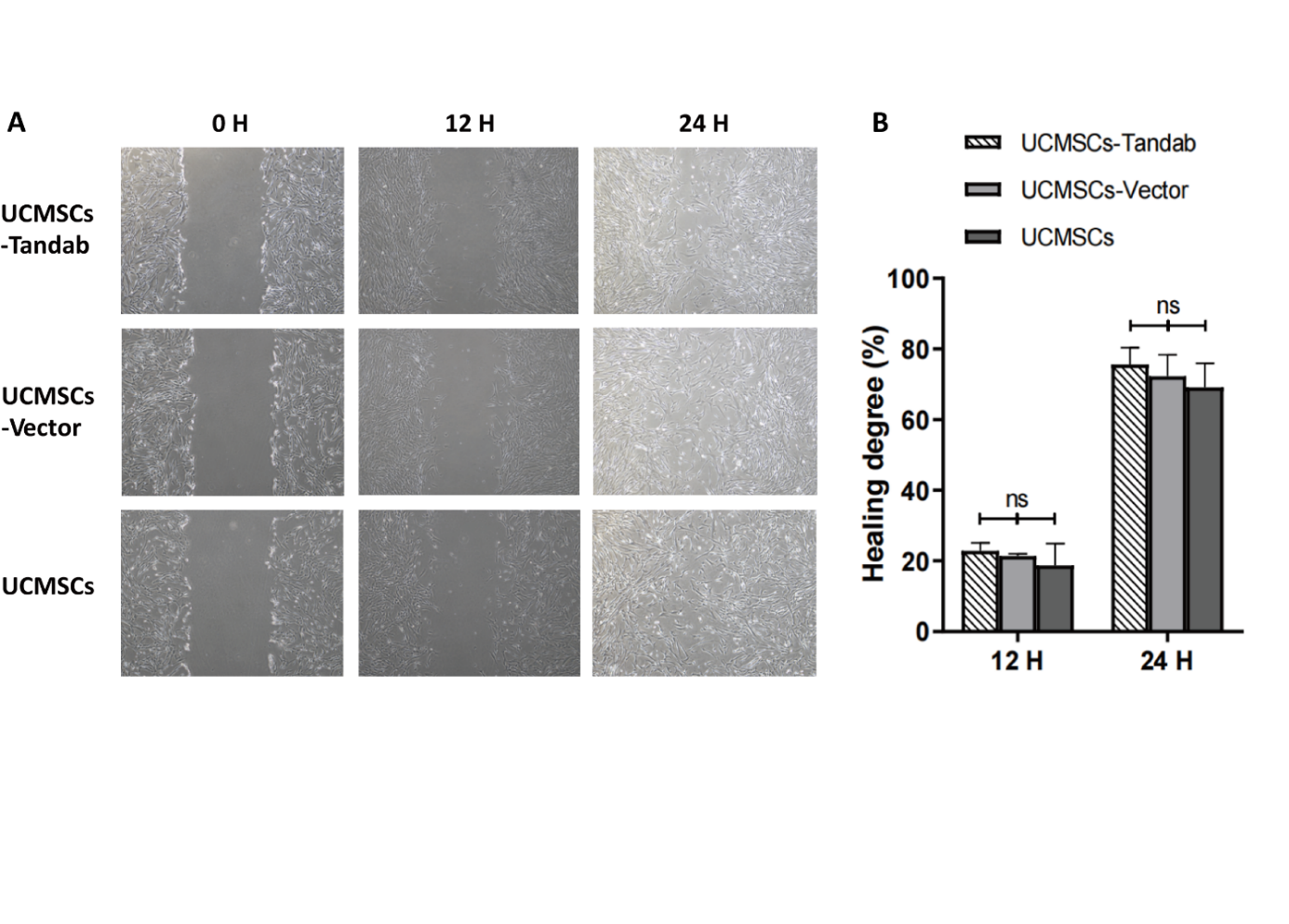


**Figure S2. The migration ability of modified UCMSCs**

1. The migration ability of UCMSCs-Tandab(IL-6/CD20), UCMSCs-Vector and UCMSCs by wound healing scratch assay. (B) The cell healing degree percentage was statistically analyzed on 12 h and 24 h, respectively. n=3 for each group, ns: not significant.
